# Foodborne viral outbreaks associated with frozen produce

**DOI:** 10.1017/S0950268819001791

**Published:** 2019-10-18

**Authors:** Neda Nasheri, Adrian Vester, Nicholas Petronella

**Affiliations:** 1 National Food Virology Reference Centre, Bureau of Microbial Hazards, Food Directorate, Health Canada 251 Sir Frederick Banting Driveway, Ottawa, ON, K1A 0K9, Canada; 2 Biostatistics and Modeling Division, Bureau of Food Surveillance and Science Integration, Food Directorate, Health Canada Ottawa, ON, Canada

**Keywords:** Frozen fruit, Hepatitis A virus, Norovirus, Outbreak

## Abstract

Over the past decade, frozen fruits have been a major vehicle of foodborne illnesses mainly attributed to norovirus (NoV) and hepatitis A virus (HAV) infections. Fresh produce may acquire viral contamination by direct contact with contaminated surface, water or hands, and is then frozen without undergoing proper decontamination. Due to their structural integrity, foodborne viruses are able to withstand hostile conditions such as desiccation and freezing, and endure for a long period of time without losing their infectivity. Additionally, these foods are often consumed raw or undercooked, which increases the risk of infection. Herein, we searched published literature and databases of reported outbreaks as well as the databases of news articles for the viral outbreaks associated with the consumption of frozen produce between January 2008 and December 2018; recorded the worldwide distribution of these outbreaks; and analysed the implication of consumption of different types of contaminated frozen food. In addition, we have briefly discussed the factors that contribute to an increased risk of foodborne viral infection following the consumption of frozen produce. Our results revealed that frozen fruits, especially berries and pomegranate arils, contributed to the majority of the outbreaks, and that most outbreaks were reported in industrialised countries.

## Introduction

The growing demand for fruits and vegetables in developed countries has led to increased importation of produce, which is often frozen to prolong shelf life. Today, the frozen food market is one of the largest sectors of the food industry, with a value of over US$75 billion in the USA and Europe combined [1]. However, frequent foodborne outbreaks and illnesses associated with imported frozen produce have raised concerns regarding the safety of these commodities.

Norovirus (NoV) and hepatitis A virus (HAV) are the leading causative agents responsible for numerous foodborne outbreaks associated with the consumption of contaminated frozen produce [2–4]. These viruses are transmitted through the faecal–oral route, and are shed in high numbers. It has been shown that one milligram of faeces from infected individuals may contain 10^6^ to 10^8^ genome copies of HAV or NoV [5, 6]. Importantly these viruses are highly infectious, as it has been estimated that only 1–10 viral particles are sufficient to infect humans [7, 8]. Therefore, contamination of foods with microscopic amounts of infected faeces can cause outbreaks and illnesses.

NoV is a non-enveloped, positive-sense, single-stranded RNA virus that belongs to *Caliciviridae* family [9]. Based on genetic diversity, NoV is divided into at least seven genogroups (G), of which viruses from GI, GII and GIV infect humans. The GI and GII NoV are further categorised into nine and 22 different genotypes, respectively, based on the sequence diversity in the complete capsid protein [10]. Over the past decade, the majority of NoV infections have been caused by GII.4 [11], but multiple genotypes are co-circulating at any given time. Subsequent to a short incubation time (12–48 h), NoV causes acute gastroenteritis that normally lasts only 2–3 days but can persist longer in immunocompromised individuals [12]. Each year, NoV has been estimated to cause 685 million cases of gastroenteritis, leading to 200 000 deaths globally [13, 14]. Due to the lack of a robust and readily available cell culture system for NoV, most of our knowledge about NoV transmission, survival and inactivation is obtained by human challenge studies or by using cultivable surrogate viruses such as murine norovirus (MNV) and Tulane virus (TV) [15]. Recent successful replication of multiple NoV strains in human intestinal enteroids (HIEs) was a breakthrough in NoV research [16, 17]. Although further development is needed to improve its efficiency, the HIE system is a promising tool for investigation of the human NoV transmission, provides a means for viral enrichment from naturally contaminated foods and can support the evaluation of inactivation mechanisms [17].

HAV is also a non-enveloped, positive-sense, single-stranded RNA virus of the *Picornaviridae* family. Subsequent to an incubation period of 15–50 days, HAV infection causes acute hepatitis that typically lasts for no more than 2 months, but some patients may demonstrate prolonged or relapsing symptoms for up to 6 months [18]. In spite of an effective vaccine, it has been estimated that millions of new HAV infections occur every year [19], which lead to about 90 000 deaths worldwide [20].

HAV has been shown to have a single conserved antigenic neutralisation site, and therefore, all isolates from different parts of the world belong to a single serotype. Despite this, HAV displays some degrees of genomic diversity, which allows for its classification into six genotypes (I–VI). Genotypes I–III have been associated with infections in humans and are further divided into two sub-genotypes (A and B). Genotypes and subtypes are often associated with different geographic distribution [21]. In general, genotype I is the most prevalent genotype, with subtype IA being more common than IB [22].

While there are several studies concerning the viral outbreaks linked to fresh produce [23, 24], there is no review study dedicated to the prevalence of viral outbreaks associated with frozen produce, especially that frozen fruits were major vehicles of foodborne viral outbreaks in recent years. The purpose of this review article is to describe the spatio-temporal distribution and the magnitude of the foodborne viral outbreaks related to the consumption of frozen fruits and vegetables over the past decade, to advance the current knowledge of the risk of foodborne illnesses associated with the consumption of these commodities.

### Methods

To identify published outbreak reports, we used the Public Health Agency of Canada database PAIFOD (Publically Available International Foodborne Outbreak Database), containing reports from January 2008 to December 2018. PAIFOD is compiled systematically from reports in peer-reviewed journals, listservs, press releases, reports from health units and government websites worldwide such as scientific literature, ProMED Digest, Eurosurveillance, etc. [25]. The PAIFOD report was prepared by searching for outbreaks of NoV, and HAV associated with frozen fruits and vegetables from January 2008 to December 2018.

News article text for the period January 2008 to December 2018 was obtained from the Global Public Health Intelligence Network (GPHIN) [26] database. The GPHIN analyses more than 20 000 online news reports in nine languages worldwide every day. The GPHIN aggregates data based on an algorithm that provides potential signals of emerging public health events, which are then reviewed by a multilingual, multidisciplinary team [26]. For the GPHIN search, the keywords: Norovirus, Hepatitis A virus, Norwalk virus, winter vomiting disease, two-bucket disease and hep A were used. In order to identify those articles that were reporting an actual outbreak, we used natural language processing. First, we identified quantitative statements using the named entity recognition implemented in SpaCy (https://github.com/explosion/spaCy). Then we used syntactic tree parsing to identify clauses involving these quantitative nouns. For example, a quantitative statement is ‘10 people’ and the clause it is part of is ‘10 people become ill’. We used a set of keywords to identify clauses, which refer to people being affected by the virus, such as ill, hospitalised, sickened, etc. Quantitative statements that were returned using this method were either specific statements about an outbreak, such as ‘on Sunday 10 people contracted norovirus’ or general statements about virus contagion, such as ‘Norovirus causes more than 20 million illnesses annually in the US’. In order to distinguish between these two categories of statements, we used regularised logistic regression. Testing in cross-validation suggested that the classifier has a highly accurate area under the ROC curve of 0.92. After identifying specific statements of virus impact on a quantitative number of individuals in each article, we combined multiple clauses across an article by taking the clause with the largest number of people.

In order to place these events onto a map for visualisation, we extracted locations from the article using named entity recognition in SpaCy. Using the Nominatim API, these location references were assigned to a country, or a state/province in the case of the USA and Canada. When there were multiple location references in an article, we preferentially used the title of the article, then the sentence of the quantitative clause and then the entire article. Within each country (or state/province for the USA/Canada), we assigned random latitude/longitude coordinates so that each article could be displayed on the map.

## Results

### Officially reported outbreaks associated with frozen fruits and vegetables

**Fig. 1.**
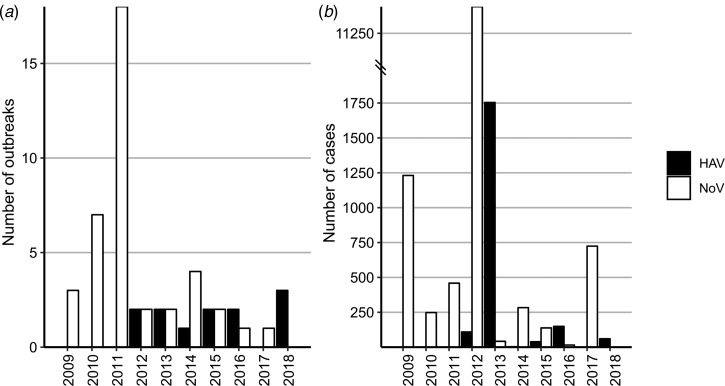
(a) The yearly distribution of the number of the reported viral outbreaks associated with frozen produce. (b) The yearly distribution of the number of the reported cases associated with HAV and NoV infections from frozen produce. Data are from the PAIFOD database and are shown for both NoV (blue) HAV (red).

We first investigated the publically available databases for the reported outbreaks associated with frozen fruits and vegetable in the past decade (2008–2018). Outbreak reports involving frozen produce identified in our search included 12 outbreaks of HAV involving 2114 cases and 40 reports of NoV involving 14 516 cases (Supplementary Tables 1 and 2). The number of cases per outbreak ranged from 2 to 1589 for HAV (mean 176) and 2 to 11 200 for NoV (mean 372). No HAV outbreaks linked to frozen fruits or vegetables were reported from 2008 to 2011, and in general the total number of NoV outbreaks associated with frozen fruits was more than three times higher than the number of outbreaks for HAV (40 individual outbreaks *vs.* 12) (Fig. 1a). NoV infections comprised a higher number of total cases over the past 10 years (Fig. 1b), except in 2013, when multiple outbreaks of HAV associated with different frozen fruits were reported in Europe and North America. For example in 2013, Italy experienced a large HAV-1A outbreak with approximately 1800 reported cases, associated with frozen berries [27]. As well as another outbreak with 165 confirmed cases of HAV-1B occurred in 2013 in the USA that was attributed to imported frozen pomegranate arils [3].

**Fig. 2.**
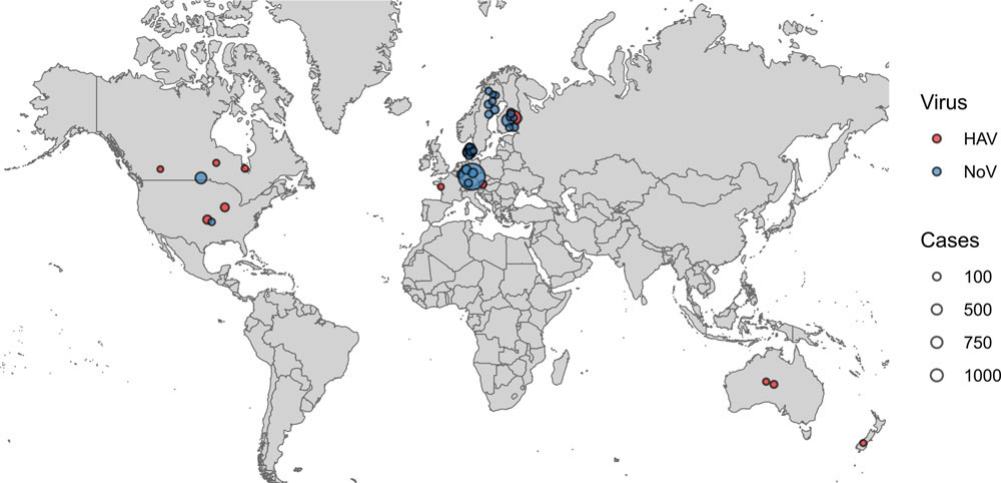
Geographical distribution of reported viral outbreaks linked to frozen fruits. Each outbreak in the PAIFOD database was mapped onto its country of origin. Points were assigned a random position within their country of origin to allow visualisation on the map. The size of each point corresponds to the number of cases in each outbreak. Blue points correspond to NoV and red points correspond to H A V.

**Fig. 3.**
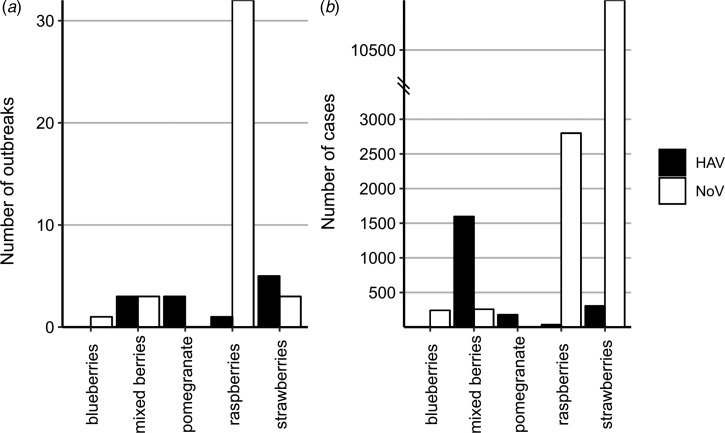
Type of frozen fruit implicated in viral outbreaks from the published sources. (a) Total number of outbreaks. (b) Total cases of illnesses. Data from the PAIFOD database were curated into one of five commonly occurring categories. Data are shown for both Norovirus (blue) and Hepatitis A (red).

HAV outbreaks associated with frozen fruits have been reported in North America, Europe, Australia and New Zealand, while NoV outbreaks linked to frozen fruits have only been reported in North America and Europe (Fig. 2). Also the vehicles for most HAV outbreaks were frozen strawberries and frozen pomegranate arils, with 41% and 25% of the total number of HAV outbreaks, respectively, whereas, frozen raspberries were indicated as the source of the majority of the NoV outbreaks (over 81% of the total NoV outbreaks) (Fig. 3a). Contaminated frozen strawberries alone were responsible for over 5000 cases of foodborne viral illnesses and contaminated frozen raspberries, caused near 3000 reported sicknesses (Fig. 3b). These numbers do not account for the total cases of illnesses associated with mixed berries, where a single source was not identified.

### Foodborne viral outbreaks reported in the news outlet

**Fig. 4.**
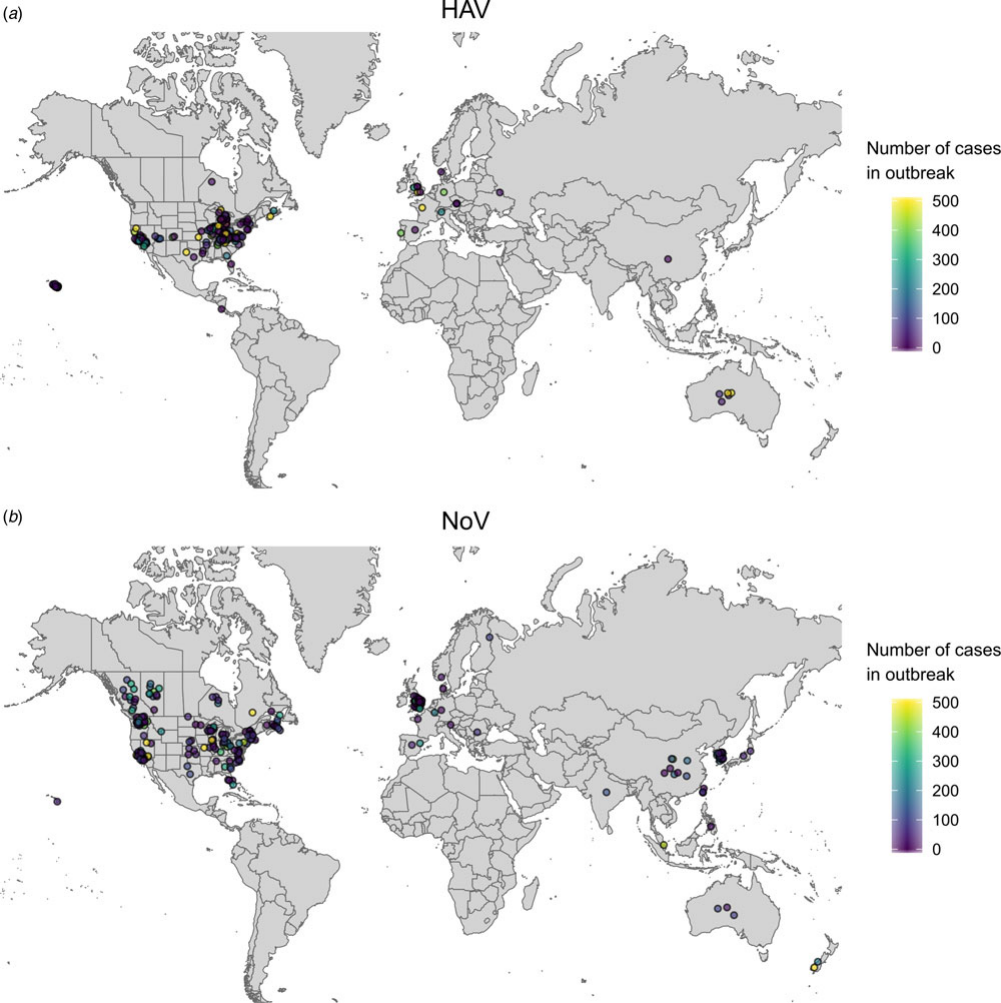
A map showing the location of newspaper articles from the GPHIN database describing outbreaks of Hepatitis A (a) and Norovirus (b). Only those articles describing a quantitative number of people becoming sick are included. The colour of the point corresponds to the number of people reported sick in each article.

To further investigate the distribution of foodborne viral illnesses associated with frozen fruits and vegetables, we consulted the GPHIN for the references to HAV or NoV from 2008 to 2018, and screened the resulting reports for relevance and content. While all the officially reported outbreaks were confirmed by the GPHIN system (Fig. 4a and b), the data obtained from the GPHIN database revealed that the number of outbreaks for both NoV and HAV is dramatically higher than indicated by official reports alone, and the outbreaks seemed to be more geographically dispersed (Fig. 4a and b). For example, HAV outbreaks in China were only found by the GPHIN system (Fig. 4a). Moreover, multiple outbreaks of NoV were identified in China, Japan, India and South Korea using the GPHIN system (Fig. 4b).

## Discussion

Control and prevention of foodborne viral illnesses particularly in fruits and vegetables is challenging, and below we will discuss the issues that to-date have hampered the foodborne viral intervention strategies.

### Mechanisms of contamination

There are several factors contributing to increased risk of viral contamination of berries and vegetables during primary production, post-harvest processing and distribution. Generally, they grow close to the ground, which increases their contacts with potential contaminants such as sewage-polluted irrigation water, or organic waste such as manure or sewage sludge used as soil fertilisers [28, 29]. Importantly, there is evidence that enteric viruses such as HAV and NoV can become internalised in berries and vegetables and therefore be protected from downstream decontamination practices [30–32]. Furthermore, berries and certain vegetables undergo multiple manipulation processes from the point of harvest to consumption, which increase the risk of their contamination by infected handlers compared to other foods [33, 34].

### Persistence and resilience of foodborne viruses

HAV, and NoV, like most other enteric viruses, are non-enveloped and possess a hardy protein capsid that protects the genome and makes them environmentally stable [35]. Therefore, these viruses can persist in food and in the environment for months without losing their infectivity. In addition, common post-harvest treatments such as washing with cold or warm water typically reduce the viral load by less than one log unit, rendering them ineffective for the removal of viral particles from berries and vegetables [36, 37].

Due to the structural integrity of the virus particles, freezing and repeated freeze–thaw cycles do not reduce HAV or NoV to levels sufficient to ensure safety. Freeze-drying of fruits and vegetables has been demonstrated to decrease the infectivity of these viruses by <2 logs [36, 38]. Therefore, a risk of infection may still exist if frozen produce contaminated with HAV or NoV are consumed without additional decontamination.

### Inactivation of foodborne viruses

Due to the nature of fresh produce and the need to maintain sensory characteristics, available decontamination methods are of limited effectiveness in deactivating foodborne viruses [29]. The efficacy of various inactivation methods against foodborne viruses has been recently reviewed comprehensively [39]. For example, one study demonstrated that UV-C treatment of fresh and frozen berries is not completely efficient in inactivation of HAV and MNV [40]. Treatment of fresh raspberries with gaseous ozone at a high dose (5 ppm for 3 min) did not reduce HAV infectivity significantly, and inactivated MNV by approximately 2 log [41]. Treatment of fresh strawberries with a combination of 0.5% levulinic acid and 0.5% sodium dodecyl sulphate resulted in 2.7 and 1.4 log reductions for HAV and MNV, respectively [42]. Gaseous chlorine dioxide treatment of fresh blueberries produced a 2 log reduction in TV infectivity [43]. Therefore, more stringent intervention methods or a combination of different inactivation methods is required to ensure the absence of infectious levels of enteric viruses in produce.

### Detection of foodborne viruses in foods

Although NoV and HAV cannot replicate in food, they can still cause illness due to high infectivity and low infectious dose. Nevertheless, most foodborne viral outbreaks cannot be prevented or intervened because the explicit links between a contaminated food and infected individuals cannot often be identified. There are two main challenges in source tracking of foodborne viral infections: the low sensitivity of current virus detection methods, and the inability of detection methods to distinguish between infectious and inactivated viral particles. Until recently, there was no internationally accepted method for the analysis of high-risk foods for enteric viruses. In 2013, the International Organization for Standardization (ISO) published a two-part technical method for the detection and quantification of HAV and NoV in food matrices [44, 45], this was later revised as ISO 15216-1:2017 [46]. While it was validated by many laboratories for virus detection and quantification, the ISO 15216 method does not address viral infectivity and does not provide the resolution required for foodborne virus outbreak investigation and source attribution. There are several genotyping tools for HAV, and NoV, such as HAVNet [47] and NoroNet [48], which can be used to determine viral genotypes based on partial genomic sequences; however, genotyping alone does not provide the resolution needed to reduce the noise in the epidemiological data in many outbreak settings. Enhanced genomic characterisation is necessary to link the clinical cases to suspicious food sources.

Whole-genome sequencing (WGS) is a promising tool for epidemiological investigation of a foodborne outbreak, including identification of related cases, source tracking and development of intervention strategies [49]. Although WGS methods have been employed on clinical samples to delineate linked NoV cases [50, 51], NoV WGS methods are yet to be developed to be applied on food, since the small number of viral particles, as well as the presence of inhibitors in naturally contaminated food products, make the current WGS protocols inefficient [52]. For this reason, even exhaustive data mining of 29 million sequence reads obtained from RNA-Seq analysis of naturally contaminated frozen strawberries led to the recovery of only one short read, with a length of 146 bp, that showed homology to the NoV genome [53]. However, for proper NoV outbreak delineation, full capsid sequence (1628 bp) is required at the minimum [54].

### Vaccine challenges

Vaccination is considered a highly effective mode of prevention and control of infectious diseases. Given the burden of the illnesses caused by HAV and NoV, implementation of effective vaccination strategies would be cost-effective and beneficial to public health [55, 56]. Although there are some promising candidates in the trial, currently there is no licenced vaccine against NoV [55].

Under current World Health Organization (WHO) guidelines, HAV vaccination is recommended for immunosuppressed patients and individuals aged ≥1 year who are travelling to countries or areas with moderate to high risk of infection (https://www.who.int/ith/vaccines/hepatitisA/en/, 2019). Therefore, HAV vaccine is not advocated for universal childhood vaccination globally [57], and due to the vaccination gap, millions of people still contract HAV [19].

### Policy and guidelines challenges

Foodborne viruses are highly infectious and food samples present a challenging matrix; therefore, sensitive methods that are capable of extracting low levels of contaminating virus for downstream molecular characterisation are needed. Nevertheless, the results of molecular analysis should be interpreted with caution; positive results merely indicate the presence of viral genome, and do not address the infectivity status of the detected virus, while negative results do not completely rule out the risk of viral contamination [52]. For these reasons, viral testing is not routinely performed in regulatory food laboratories worldwide [58].

There are limited data regarding the overall prevalence of enteric viruses in fresh and frozen produce globally, a recent study by Cook *et al.* has demonstrated that the prevalence of NoV in fresh and frozen raspberry marketed in the UK is 2.3% and 3.6%, respectively [59]. Also, 8.3–36% of the tested fresh produce in Mexico were positive for HAV and NoV, respectively [60]. Furthermore, 8.6% of the tested frozen raspberries in the Netherlands were positive for NoV [61]. Since freezing does not have any significant effect in reducing viral infectivity, if contaminated fruits undergo freezing procedures, the risk of viral infection is still in place. Therefore, it can be assumed that the available data drastically underestimate the prevalence of foodborne viral illnesses associated with frozen produce.

In this review, we investigated the prevalence of the foodborne viral outbreaks attributed to frozen produce comprehensively. Despite comprehensive search through the outbreak report databases, we did not identify any foodborne viral outbreaks attributed to frozen vegetables. This finding could be partially explained by the implementation of pre-consumption processing such as cooking or steaming of the frozen vegetables by the consumers, while frozen fruits are often consumed raw.

Both the total number of outbreaks and the total number of cases associated with NoV infection were significantly higher than with HAV infection. This difference can be explained by the long-lasting HAV immunity after infection or vaccination, a high prevalence of asymptomatic HAV infections and the long HAV incubation period, which makes the outbreak identification and source attribution challenging.

While contaminated frozen strawberries and raspberries were implicated in both HAV and NoV outbreaks, contaminated pomegranate arils were responsible for three separate HAV outbreaks. Whether HAV can survive longer on pomegranate arils, or there are epidemiological factors that make them more prone to HAV contamination needs further investigation.

It has been estimated that about 65% of the world's first news about infectious disease events comes from informal sources, such as the Internet news, and the majority of large-scale outbreaks investigated by the WHO are first reported by these sources [62, 63]. For this reason, we investigated the GPHIN database for indications of the foodborne viral illnesses associated with frozen fruits and vegetables. The GPHIN system is complementary to the published outbreak reports, and its news articles have the potential to be an early indicator of the clusters of illnesses related to a foodborne outbreak. The apparent discrepancy in the number and geographical distribution between the published data and the data obtained from the GPHIN system might be partly explained by under-reporting of the foodborne viral illnesses. Under-reporting is a widely accepted issue with regards to illnesses caused by foodborne viruses. For example, a study revealed that at least 76% of NoV infections are not reported in Germany [64], and it has been demonstrated that the young and otherwise healthy individuals, when infected by these viruses, are less likely to seek medical care and thus not be properly diagnosed [65]. Another limitation of this work is the absence of data regarding the sporadic foodborne viral diseases, because, there is virtually no system to quantify the degree of sporadic transmissions. Additionally, strong epidemiological evidence is often missing to confidently distinguish the foodborne viral transmissions from the secondary person-to-person transmissions in outbreak investigations.

In this work, we searched both global databases and news outlets, and found that the majority of the outbreaks were reported in industrialised countries, and that the illnesses were mostly associated with imported frozen fruits. The discrepancy between reports of foodborne viral illness associated with frozen produce between importing and exporting countries may reflect differences in systems for outbreak investigation and surveillance [24]. Additionally, there may be differences in consumption patterns between exporting and importing countries, produce from exporters may be more likely consumed fresh, instead of frozen, or produce that is produced for export may not be consumed locally.

Recently, a systematic review article investigated the prevalence of foodborne viral outbreaks associated with fresh produce in official databases (PubMed/Medline, Scopus, Eurosurveillance Journal, Spingerlink electronic journal, ProMED-mail) up to 2016 [24]. While their major focus was on fresh produce, it was found that contaminated frozen berries were responsible for 42.8% of all the reported outbreaks linked to produce. The present review focuses on outbreaks associated with frozen produce in the past decade (2008–2018), and in addition, the GPHIN database was searched for foodborne viral outbreaks during that period. Both works revealed that most outbreaks are reported in industrialised countries with similar geographical distribution [24], NoV was responsible for the majority of outbreaks, and soft fruits and berries were implicated as frequent vehicles of infection [24].

Altogether, the current review demonstrated that viral contamination of frozen produce leads to numerous outbreaks around the world. Since conventional inactivation techniques are not effective in virus inactivation [39], new pre- and post-harvest processing technologies should be assessed for their viricidal potential in high-risk foods such as fresh and frozen produce [66]. It is also recommended that preventative measures, such as effective hand and environmental decontamination procedures [67], should be taken into consideration to reduce the risk of contamination with foodborne viruses [68]. Thus, strengthening safety measures in the production of fruits and vegetables could be an effective way to prevent foodborne viral infections. For example, protection of irrigation water from faecal pollution should be considered as a goal for prevention of contamination within production systems [15, 29]. In addition, implementation of stringent policies, such as the Food Safety Modernization Act (FSMA) [69], which contains regulations for raw agricultural commodities, such as specific fruits and vegetables, are required to ensure the virological safety of produce. Finally, regular monitoring activities should be conducted to provide information about potential virus contamination using suitable indicators of viral contamination or reliable foodborne virus detection methodologies [66]. Intervention strategies should be considered upon the identification of viral contamination, combined with a formal international requirement to report outbreaks and contaminations to a central agency to improve early detection of international sources of viral outbreaks.

## Summary and conclusions

The search in the official databases led to the identification of 12 HAV outbreaks involving 2114 cases and 40 NoV outbreaks involving 14 516 cases related to the consumption of frozen fruits. The GPHIN data, however, suggest that the reported number of outbreaks could be an underestimation. Foodborne viral outbreaks remain a major public health concern, and controlling them requires guidelines specifically aimed at the reduction of viral contamination in produce at pre- and post-harvest stages, and regular surveillance by the public health bodies [66]. Furthermore, sensitive viral extraction methodologies are required for both genomic characterisation and assessment of infectivity, and corrective actions should be implemented once sources of contamination are identified [66, 68]. Finally, the GPHIN system has the potential to be used to aggregate the information required to determine trends and outbreak hotspots in foodborne illnesses.

## Supplementary material

For supplementary material accompanying this paper visit https://doi.org/10.1017/S0950268819001791. click here to view supplementary material
